# The Effect of Reinforcement Preheating Temperatures on Tribological Behavior of Advanced Quranic Metal-Matrix Composites (QMMC)

**DOI:** 10.3390/ma15020659

**Published:** 2022-01-16

**Authors:** Sultan Althahban, Yosef Jazaa, Omar Bafakeeh, Abdullah S. Alomari, Hossam El-Din M. Sallam, Mahmoud Atta

**Affiliations:** 1 Department of Mechanical Engineering, Jazan University, Jazan 82822, Saudi Arabia; smalthahban@jazanu.edu.sa; 2 Department of Mechanical Engineering, King Khalid University, Abha 61421, Saudi Arabia; yosef.jj@gmail.com; 3 Department of Industrial Engineering, Jazan University, Jazan 82822, Saudi Arabia; albafakeeh@gmail.com; 4 Nuclear Science Research Institute, King Abdulaziz City for Science and Technology (KACST), Riyadh 11442, Saudi Arabia; asalomari@kacst.edu.sa; 5 Materials Engineering Department, Zagazig University, Zagazig 44519, Egypt; 6 Mechanical Design and Production Engineering Department, Zagazig University, Zagazig 44519, Egypt; matta767@gmail.com

**Keywords:** copper matrix composite, tribological behavior, interface bonding, mechanical properties, manufacturing parameters

## Abstract

The growing applications of iron/copper bimetallic composites in various industries are increasing. The relationship between the properties of these materials and manufacturing parameters should be well understood. This paper represents an experimental study to evaluate the effect of reinforcement (steel rod) preheating temperature on the mechanical properties (bond strength, microhardness, and wear resistance) of copper matrix composites (QMMC). In preparing the QMMC samples, the melted copper was poured on a steel rod that had been preheated to various temperatures, namely, room temperature, 600 °C, 800 °C, and 1200 °C. Properties of the QMMC (interface microstructure, interfacial bonding strength, microhardness, and wear) were investigated. The experimental results revealed that the best bond between the copper matrix and steel rod formed only in the composites prepared by preheating the steel rods with temperatures lower than the recrystallization temperature of steel (723 °C). This is because the oxide layer and shrinkage voids (due to the difference in shrinkage between the two metals) at the interface hinder atom diffusion and bond formation at higher temperatures. The microhardness test showed that preheating steel rod to 600 °C gives the highest value among all the samples. Furthermore, the QMMC’s wear behavior confirmed that the optimization of preheating temperature is 600 °C.

## 1. Introduction

Copper (Cu)-based materials are an important class of materials because of their good machinability and excellent electrical and thermal properties, making them suitable for a wide range of applications [1]. However, for many applications, pure Cu and its alloys cannot be used because of their relatively low hardness, low strength, as well as inferior tribological properties [2]. Moreover, they are easily deformed under heavy loads or in friction and wear processes. Therefore, improving the mechanical properties of Cu-based materials while maintaining excellent electrical and thermal conductivities becomes indispensable for their utilization in cutting-edge technological applications, including high precision navigation instruments, electrical systems, data communications, and air conditioning [3,4,5,6,7].

Metal matrix composites (MMCs) technology offers the possibility of customizing properties of Cu-based materials. In this technique, a ceramic material such as SiC, Al_2_O_3_, and TiC is embedded into the copper matrix. The parameters controlling the choice of reinforcements in copper matrix composites are the compatibility, chemical and thermal stability, availability, and cost of processing and production [8,9,10,11]. Cu naturally has poor wettability with ceramic materials, resulting in weak interfacial bonding and high thermal resistance. The inferior interface bonding is detrimental to both the mechanical and physical properties of copper matrix composite [12]. In recent decades, several approaches, including coating with wetting agents and chemical and heat treatments, have improved the interface bonding between Cu matrices and ceramic reinforcements. Although those approaches showed promising improvements in the properties of Cu matrix composites, some of them are relatively complex and expensive [13,14,15,16]. However, using hard metallic reinforcements instead of ceramic materials is considered a viable approach. Cu matrix composites can be designed with superior mechanical and tribological properties due to their adequate wettability with metal matrices [17].

Iron alloys, especially steels, are an attractive choice for metallic reinforcement because of their high mechanical properties, abundance, and reasonable costs for processing and production [18,19,20]. For example, Alaneme and Odoni [21] compared the mechanical and tribological behavior of stir-cast Cu matrix composites reinforced with steel machining chips (SMC) with those reinforced with Al_2_O_3_ particles. The results showed that mechanical (hardness and tensile strength) and tribological properties of Cu composites reinforced with SMC were higher than that of Cu composite reinforced with Al_2_O_3_ particles. Improvement in properties was attributed to the strong Cu/SMC interface, which facilitates load transfer from the Cu matrix to the strong SMC reinforcements. In recent work by Zhang et al. [22], tribological behaviors of copper-based composites were investigated. They concluded that iron shows better particle strengthening due to the formation of the diffusion bonding interface between iron and the copper matrix. However, the manufacturing method used to prepare such composites affects the strong interface bonding between the copper matrix and steel reinforcements. In previous work by our group, an experimental and numerical study was conducted to investigate the mechanical and bond behavior of the copper matrix composite reinforced with a steel rod [23].

The origin of the present research idea is stimulated from the story of Alexander’s Iron Gates or the wall of Gog and Magog [24]. Two verses from the Holy Quran more than 1400 years ago reported the preheating of reinforcement to obtain good properties in a bimetallic composite [25]; more details about this interesting story can be obtained in the references [24,25]. Recently, Sallam et al. [26,27] suggested preheating the austenitic stainless steel reinforcements to about 700 °C to reduce temperature losses in the bimetallic casting process. Kang et al. [28] and Norouzifard et al. [18] also found that the preheating of reinforcements positively affects the final properties of copper composites. Based on these points, the present work’s main objective was formulated. The main objective is to study the effect of the steel pieces preheating temperature on the tribological behavior of Cu/steel bimetallic composites. It is worth noting that the authors previously conducted a preliminary study [23] to choose the best procedure for adding copper to steel based on the bond strength between them. Therefore, the challenge of the present research is to get the best preheated temperature from the following temperatures: room temperature, 600 °C, 800 °C, and 1200 °C.

## 2. Materials and Methods

### 2.1. Materials and Sample Preparation

The present experimental work used commercially available copper wires with 99.5 wt.% purity and mild steel rods with a nominal diameter of about 6 mm to fabricate QMMC samples. The chemical composition of copper and steel is shown in Table 1. 

**Figure 1:**
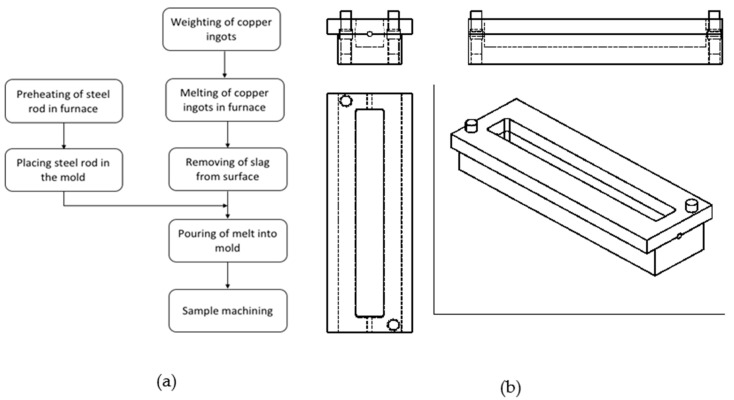
(**a**) Flowchart of foundry process used to prepare QMMC; (**b**) D2 steel mold used to make QMMC samples.

The copper matrix composite with steel rods as reinforcements was fabricated using the foundry processes, as shown in Figure 1a. An amount of 2 kg of highly pure copper charge was melted at 1125 °C in a resistance furnace. During the melting process of the copper, a mild steel rod was cleaned and polished to remove any contaminations that might exist on its surface and interfere with the result. There were four different trials; in each trial, the temperature of the steel rod was different: room temperature, 600 °C, 800 °C, and 1200 °C. 

Subsequently, after removing the dross from the surface, the copper melt was poured into the mold containing the steel rod (Figure 1b). Finally, the prepared composite specimens were machined to obtain the required dimensions for each test.

### 2.2. Microstructural Characterization

Microstructural examination of the QMMC samples was performed using optical microscopy and scanning electron microscopy (SEM). Optical microscopy (MT5210 MEIJI Techno, Japan) was utilized to investigate the microstructure and surface morphology of the QMMC samples. The samples were prepared for metallographic examinations with a series of grinding and polishing operations. For a detailed study of the microstructural features and elemental composition of the composites produced, a field emission gun scanning electron microscope (JEOL JSM-6380 LA) at an accelerating voltage of 25 kV was used. The SEM instrument is fully embedded with an energy dispersive X-ray spectrometer (EDS) that was employed to quantitively map the element distribution profile of the interface between the steel rod and copper matrix of the QMMC samples through Oxford Aztec software (OAS). The step size in the interval between each sampling point along the EDS line mapping was 0.2 µm. MS Excel was used to generate the final EDS spectra using data collected from OAS. Before performing micrography in the SEM, the samples were ultrasonically cleaned in acetone for 60 min.

### 2.3. Push-Out Test

**Figure 2:**
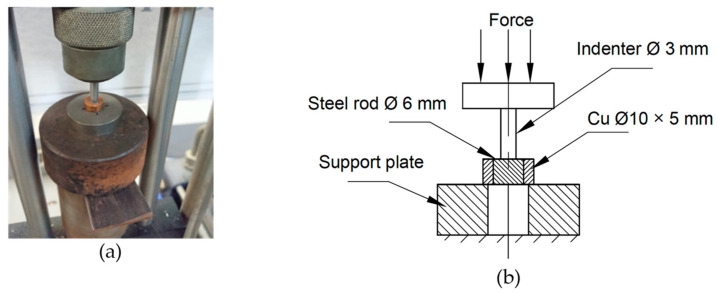
(**a**) Photograph of the actual push-out test set up; (**b**) diagrammatic sketch of the push-out test set up.

This test is performed to measure the matrix/fiber interface bonding strength between the copper matrix and the steel rod, which serves as reinforcement. A cylindrical sample (Ø 10 mm × 5 mm) was made from each Cu/Fe composite. The push-out test was carried out on a universal testing machine according to the ASTM F1820 standards, where the steel rod was mechanically pushed out of the matrix material (Figure 2).

### 2.4. Microhardness Test

The microhardness test was conducted using a universal hardness tester (TH722, Beijing TIME High Technology Ltd.) according to the ISO 6507 (Vickers hardness test method) standards. Four cylindrical specimens were cut with a diameter of 1 cm and a height of 1 cm from the original Cu/Fe composites. These samples’ surfaces were then subjected to a series of cleaning and polishing operations until achieving a smooth and contamination-free surface. This step is essential for accurate hardness measurements. However, the hardness measurements were collected from three regions: the copper matrix, the steel rod, and the copper/steel interface. In each region, the hardness was determined at four different locations by applying a load of 30 kg for a dwell time of 10 s. After that, the mean hardness was calculated by the average of four indentations. 

### 2.5. Wear Test

**Figure 3:**
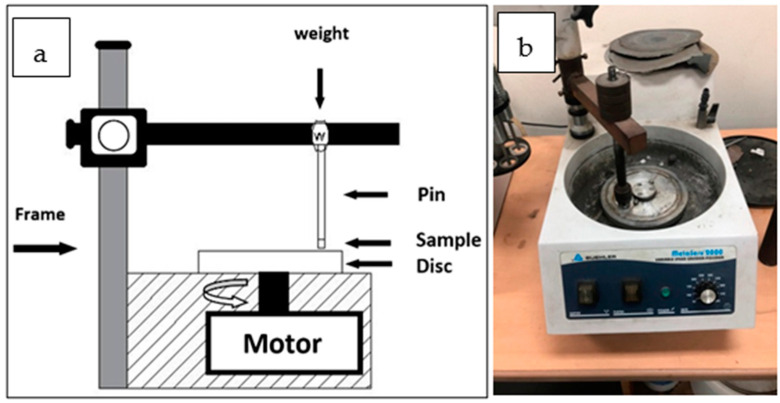
(**a**) Schematic view of the custom-built tribometer used in wear test; (**b**) photograph of the custom-built tribometer used in wear test.

The wear test was conducted using a custom-built tribometer according to the ASTM G99-95a standards (Figure 3). A cylindrical specimen (Ø 10 mm × 10 mm) from each Cu/Fe composite was tested against a GP-154 silicon carbide disc of 300 grit. A typical load of 10N was applied to the specimen, and then the SiC disc used as the abrasive counterface was rotated. Two different experiments were carried out to study the wear rate of the QMMC samples. The first one was done to study the effect of change in contact speed, while the second was conducted to study the effect of the contact distance. In the first experiment, the SiC disc was operated at four different speeds (22, 44, 88, and 176 m/min) for a fixed time (5 min). In the second one, the disc was moved at a constant speed (22 m/min) for four different distances (88, 132, 176, and 220 m). The average wear amount was measured by weight loss of the specimen (weighed by a balance with 0.1 mg precision), and the wear rate was computed according to Equation (1) [2,29]:

Wear rate = W/(ρ D)    (mm^3^/m) (1)

where ρ is the density of the material (g/mm^3^), W is the weight loss (g), and D is the sliding distance (m).

## 3. Results and Discussion

### 3.1. Microstructural Characterization

**Figure 4:**
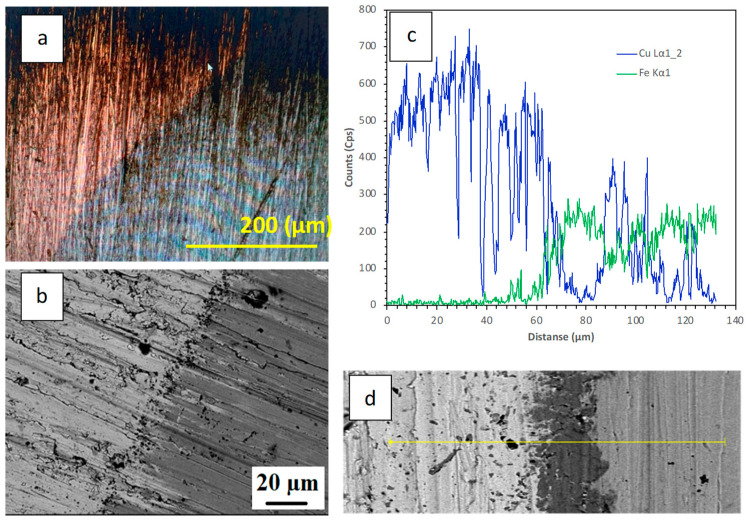
(**a**) Light microscopy image; (**b**) SEM image of a QMMC prepared by pouring molten copper onto a steel rod at room temperature; (**c**,**d**) an EDS spectrum collected from the interface region and corresponding high magnification SEM image.

Microscopy results illustrate that the bond characteristic between the copper matrix and the steel rod (reinforcement) varies with the steel rod preheated temperature (from room temperature to 1200 °C). Figure 4 depicts the microstructure of the bonding interface on QMMC prepared by pouring molten copper on a steel rod at room temperature. It reveals that the copper matrix has an adequate bond with the steel rod. Additional energy-dispersive X-ray spectroscopy(EDS) shown in Figure 4c indicates that atomic diffusion occurs across the interface, but the thickness of the diffusion layer is less than 20 µm due to the rapid solidification process. It is confirmed that the metallurgical bonding between the copper matrix and the steel rod is obtained [22]. Higher magnification SEM images, as shown in Figure 4b,d, demonstrate that the interface contains some microvoids that are linked to solidification shrinkage [30].

**Figure 5:**
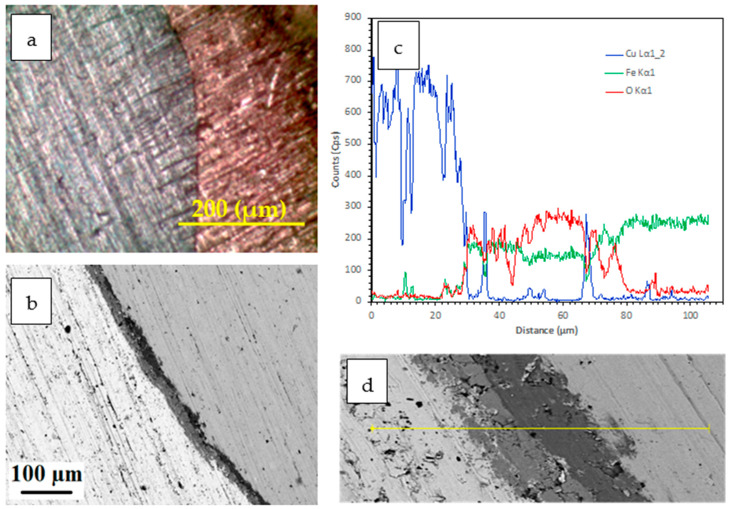
(**a**) Light microscopy image; (**b**) SEM image of a QMMC prepared by pouring molten copper onto the steel rod at 600 °C; (**c**,**d**) an EDS spectrum collected from the interface region and corresponding high magnification SEM image.

**Figure 6:**
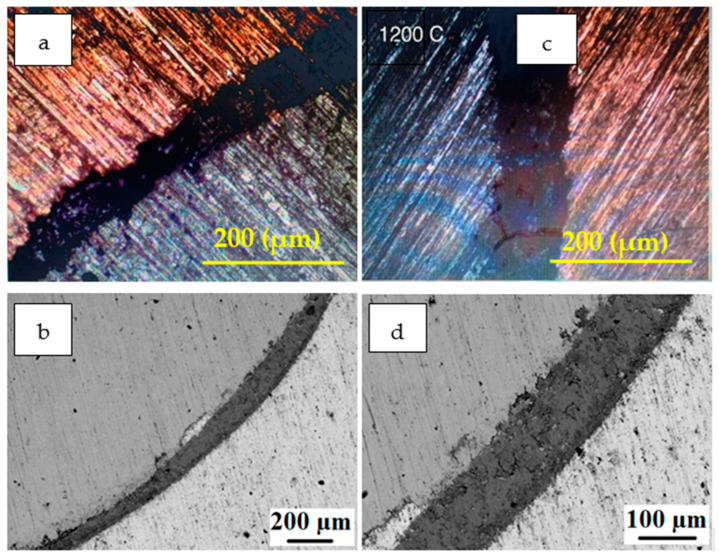
(**a**) Light microscopy image; (**b**) SEM image of a QMMC prepared at 800 °C; (**c**) and (**d**) are light microscopy and SEM images, respectively, of QMMC prepared at 1200 °C.

**Figure 7:**
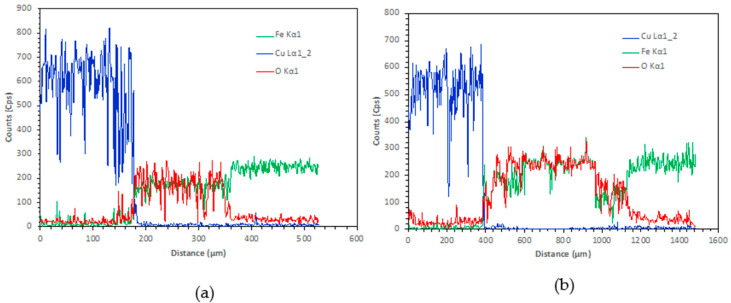
Energy-dispersive X-ray spectroscopy (EDS)of QMMC prepared at (**a**) 800 °C and (**b**) 1200 °C.

Figure 5, Figure 6 and Figure 7 show the microscopic results of the copper matrix reinforced with a preheated steel rod. Figure 5 presents an exemplary microstructure of QMMC prepared by pouring molten copper onto the steel rod at 600 °C. As revealed by optical and SEM images (Figure 5a,b,d), the interface has a discontinuous thin (about 11 μm) oxide layer containing micro-cracks and voids. This layer could hinder the bonding between the copper matrix and the steel rod [31]. The EDS profile collected from the interface region (Figure 5c) shows peaks of copper (Cu), iron (Fe), and oxygen (O), confirming the matrix-reinforcement bonding and the oxidation of the reinforcement’s surface. As seen in Figure 6, the thickness of the oxide layer increases steadily with temperature. The thickness of the layer increased from 66μm at 800 °C to about 135 μm at 1200 °C. The EDS spectra shown in Figure 7 did not show any evidence of the atomic diffusion at the interface. In other words, the EDS analysis demonstrates that the oxide layer acts as a barrier for the metallurgical bonding between the copper matrix and the steel rod. 

### 3.2. Interfacial Bonding Strength

**Figure 8:**
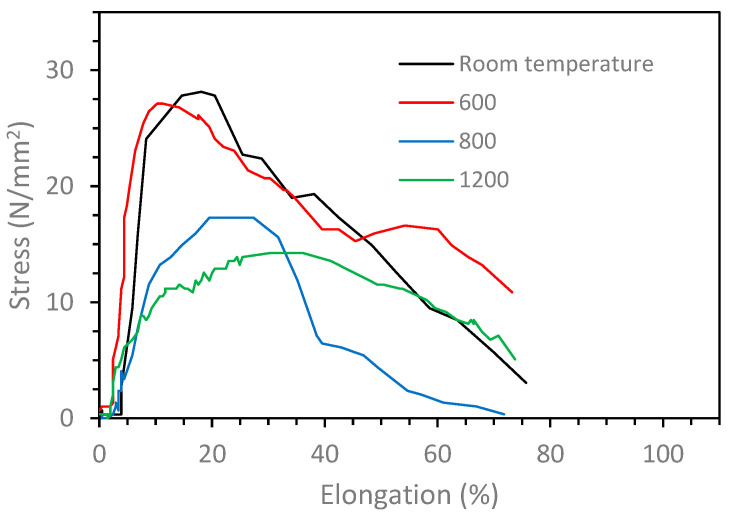
Interfacial bonding strength for the different trials of the preheating of steel rods.

The interfacial bonding strength between the steel rod and the copper matrix in the four QMMC samples was analyzed using a push-out test illustrated in Figure 2. An indenter pushes directly onto the steel rod during the push-out test, while the surrounding copper matrix is supported to minimize bending loads. The indenter diameter is smaller than the steel rod diameter (3 and 6 mm, respectively) to avoid friction between the indenter and the matrix. From the results shown in Figure 8, no significant difference was noted between the QMMC prepared at room temperature and that preheated at 600 °C. The bonding strength was about 28 ± 2 (N/mm^2^) in both cases. At higher temperatures, due to the oxide layer formation at the interface (without diffusion between Cu and Fe), the bonding strength decreased by about 40% (17 N/mm^2^) at 800 °C and 50% (14 N/mm^2^) at 1200 °C. These results are consistent with the microscopy results.

This reduction in the bonding strength can also be attributed to the shrinkage of both materials (copper and steel). The thermal strain of the used steel was given by Equation (2) and referred to the length at 20 °C [32], and the linear shrinkage allowance of copper is 2.2% [23]. This eliminated the interference between the copper (matrix) and the steel (reinforcement) from 0.15 mm (at room temperature) to less than 0.05 mm (at 1200 °C), which decreased the bonding strength between the copper and steel rod:

∆l/L = −2.416 × 10^−4^ + 1.2 × 10^−5^θ + 0.4 × 10^−8^θ^2^    20 ≤ θ ≤ 750 (2a)

∆l/L = 11 × 10^−3^    750 < θ ≤ 860 (2b)

∆l/L = −6.2 × 10^−3^ + 2 × 10^−5^θ     860 < θ ≤ 1200 (2c)

where ∆l is the elongation, L is the length at 20 °C, and θ is the steel temperature (°C).

While at 600 °C, in spite of the presence of O_2_ (likely attributable to thepresence of iron oxide) the diffusion between Cu and Fe gave a slightly higher bonding strength than room temperature, where O_2_ was not found.

### 3.3. Hardness Measurements

**Figure 9:**
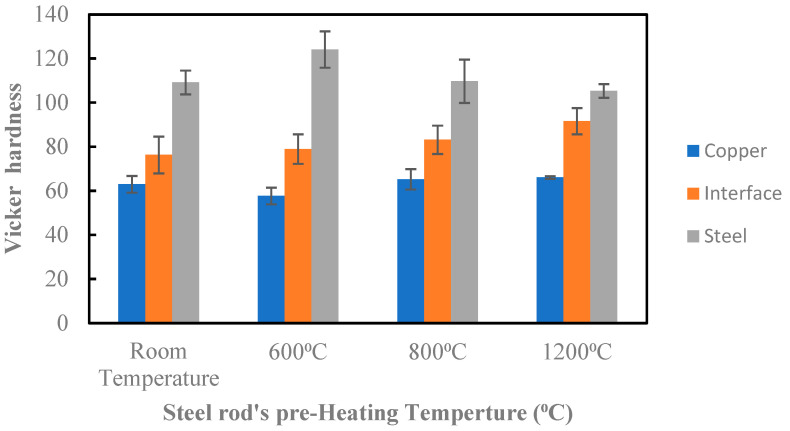
Vickers hardness of copper, interface, and steel rod at various preheated temperatures of steel rod.

Figure 9 shows the resulting average Vickers hardness of the copper, the interface and the steel rod, and the different preheating temperatures of the steel rods. For the interface region, the hardness was increased with the increase in preheating temperature. This could be due to the increase in iron oxide formation with the increase in the preheating temperature.

For the steel rods, the highest hardness was obtained at 600 °C and then decreased as the preheating temperature increased. For the preheating temperatures of 800 °C and 1200 °C, the steel rod went into full annealing heat treatment (the temperatures of the steel rod’s temperatures were greater than the upper critical temperature, austenite zone). This resulted in softening the steel rods. At 1200 °C, the steel rods stayed in the austenite zone for a longer period of time than when the temperature was 800 °C, and this reduced its hardness. At 600 °C, the steel rods undergo a subcritical or intercritical annealing heat treatment (their temperature was either below the lower critical temperature or slightly above it). The austenitizing time was short, and the small amount of austenite that had formed decomposed rapidly as it transformed into a harder material. This material is a mixture of carbide and ferrite (a significant length of time is required for it to completely transform into ferrite and pearlite) [33]. However, in all of the QMMC samples, no clear trend was noticed in the microhardness of the copper matrix.

### 3.4. Wear Analysis

**Figure 10:**
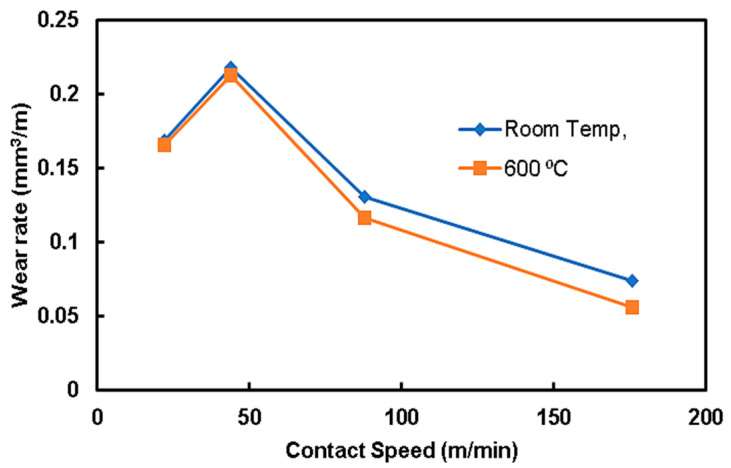
The wear rate of Cu/Fe composite (QMMC) against the contact speed with the abrasive disc.

**Figure 11:**
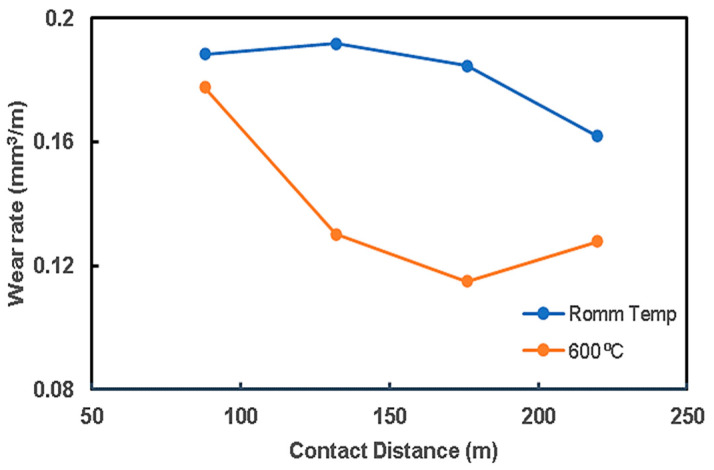
The Cu/Fe composite (QMMC) wear rate against the contact distance with the abrasive disc.

The previous results show that the preheating operation of steel rods to 800 °C and 1200 °C weakened the composites. Therefore, the wear analysis was restricted to two trials of preheating steel rods: room temperature and 600 °C, which gave the highest bonding strength. Figure 10 shows the relationship between the wear rate of the QMMC (at room temperature and with preheating the steel rod to 600 °C) and the contact speed with the abrasive disc. Preheating the steel rods to 600 °C improves the wear resistance of the composite, and this improvement is increased with the increase in the contact speed. This enhancement began with about 2% for a speed of 22 m/min to 24% at 176 m/min. Figure 11 shows the relationship between the wear rate of the same trials QMMC and the contact distance with the abrasive disc. The figure indicates that the preheating of the steel rods to 600 °C improves the wear rate resistance of the composite. This improvement is increased by increasing the contact distance to 176 m, then reduced to a smaller percentage. The enhancement began with about 6% at a contact distance of 88m, increased to 38% at 176m, and then reduced to 21% at 220 m. Figure 4c,d and Figure 5c,d show diffusion of iron within the copper and vice versa, while only Figure 5c (preheated 600 °C) showed the presence of O_2_ in the shared area between Cu and Fe. This means iron oxide was likely to be formed in this area. This could be the reason for improving the 600 °C trial over the room temperature trial.

## 4. Conclusions

Microstructural characterization, interfacial bonding strength, and wear rate test analysis for the Cu/Fe metal matrix composite (QMMC) support the following conclusions:

1. The diffusion between Cu and Fe occurred at the contact interface at a temperature of 600 °C and below.
2. Preheating the steel rod up to 600 °C improved the wear resistance and slightly improved bonding strength.
3. The improvement percentage of wear rate of QMMC increased with the increase in contact speed between it and the abrasive disc.
4. The improvement percentage of wear rate of QMMC increased with the increase in contact distance between it and the abrasive disc up to 176 m, and then the percentage is decreased.
5. Preheating the steel rod to more than 600 °C reduced the bonding strength between the steel rods and copper matrix.
6. Oxidization and shrinkage of Cu and Fe prevent the diffusion between Cu and Fe at higher temperatures.
7. QMMC wear resistance increased as the steel rod’s hardness increased. In the present work, the highest steel rod hardness was obtained when heated to 600 °C.

## Figures and Tables

**Table 1:** Typical chemical compositions of the copper matrix and the mild steel rod (in wt.%).

Constituent	Cu	Fe	C	Si	Ni	Mn	P	S
Matrix (copper)	>99.5	-	-	-	0.02	Trace	<0.003	-
Fiber (steel ST37)	-	Bal.	<0.005	0.35	-	0.35	<0.04	<0.04

## Data Availability

Data sharing is not applicable.
